# Energy and Protein Intake in Mild-Moderate COPD Patients

**DOI:** 10.1007/s00408-026-00876-0

**Published:** 2026-03-18

**Authors:** Róisín Cullen, Kirtana Jagadeesh Nayak, Dave Singh, Augusta Beech, Avni Vyas

**Affiliations:** 1 Manchester Metropolitan University, Manchester, UK; 2 Medicines Evaluation Unit, An IQVIA business, Manchester, UK; 3 University of Manchester, Manchester, UK

**Keywords:** Chronic obstructive pulmonary disease (COPD), Malnutrition, Nutritional intake, FFMI

## Abstract

**Background:**

Malnutrition is often overlooked as an extrapulmonary comorbidity in chronic obstructive pulmonary disease (COPD) and there is limited information regarding the nutritional status of younger individuals with mild-to-moderate disease in outpatient settings. We assessed energy and protein intakes in mild-to-moderate COPD patients compared to a group of healthy non-smoking individuals (HNS).

**Methods:**

COPD patients and HNS were recruited; anthropometric measurements were assessed via BMI and bioelectrical impedance, while nutritional intake was assessed using a 7-day food diary and the EPIC-Norfolk Food Frequency Questionnaire (FFQ). Low fat-free mass index (FFMI) was defined as (a) < 15 kg/m^2^ for females and < 17 kg/m^2^ for males (Schols in Eur Respir J 44:1504–1520, 2014) or (b) adjusted for age, sex and BMI (Wei et al. in Front Endocrinol 14:1185221, 2023).

**Results:**

21 COPD participants and 15 HNS were recruited with a median age of 58.0 and 57.0, respectively [IQR: 55.0–63.0 and 50.0–60.0]. A significantly greater proportion of individuals with COPD had a low FFMI when using age-sex-BMI adjusted criteria (23.8 vs. 0.0%, *p* = 0.04). Using the estimated requirement threshold of 75%, more COPD patients did not meet nutritional protein requirements HNS (35% vs. 0%, respectively, *p* = 0.01).

**Conclusions:**

Our findings suggest a potential opportunity for dietary intervention in younger individuals to prevent future sequalae of malnutrition in COPD.

## Introduction

Chronic obstructive pulmonary disease (COPD) is a heterogeneous lung condition, characterised by respiratory symptoms, due to abnormalities of the airways and alveoli that cause persistent, often progressive airflow limitation [1]. COPD often co-exists with other diseases that may alter and complicate the disease trajectory. Comorbidities can develop as a consequence of having COPD or due to shared risk factors such as ageing, smoking and physical inactivity [1]. Extrapulmonary comorbidities in COPD commonly include cardiovascular disease and metabolic syndrome [1]. Malnutrition is often overlooked as an extrapulmonary comorbidity in COPD, and encompasses both under nutrition (being underweight or weight loss) and over nutrition (being overweight or obese) [2].

Muscle wasting and weight loss are common in COPD patients with severe airflow obstruction [3]. Sedentarism, appetite change, oxidative stress, inflammation and tissue hypoxia are factors that can cause sarcopenia in COPD patients [3]. While dyspnoea often limits physical activity in COPD, sarcopenia and cachexia can also contribute [3]. Low fat-free mass ((FFMI); representative of skeletal muscle mass) and low BMI are associated with worse outcomes, including increased mortality risk [4].

COPD patients have an increased basal metabolic rate and therefore require increased energy consumption to avoid weight loss [2]. Furthermore, total muscle mass is dependent on the balance between protein synthesis and protein degradation; greater degradation occurs in cachectic COPD patients [2]. Ensuring adequate nutritional intake encompassing both energy and protein requirements is essential to maintain skeletal muscle mass and weight. A systematic review of malnutrition in COPD estimated the prevalence to be 30%, with variability depending on the measurement tool used to assess nutritional status [5]. Malnutrition was higher in an inpatient setting, in individuals with more severe disease (Global Initiative for Chronic Obstructive Lung Disease (GOLD) III-IV [1]) and during exacerbations [5].

There is limited information regarding the nutritional status of younger, mild-to-moderate COPD in outpatient settings. Individuals with less severe COPD may benefit from nutritional intervention earlier in the natural history of the disease, to support either over- or under- nutrition. We investigated energy and protein intakes in a cohort of mild-to-moderate COPD patients in an outpatient setting compared to a group of healthy non-smoking individuals (HNS), while also investigating body composition and functional impairment.

## Methods

### Subjects

COPD patients and HNS were recruited from the Medicines Evaluation Unit (Manchester University NHS Foundation Trust). Participants who were ≥ 40 years old with no history of chronic systemic inflammatory disease were included. HNS had no history of respiratory disease, self-reported a smoking pack year history < 1 and normal spirometry, with a forced expiratory volume in 1 s (FEV_1_)/ forced vital capacity (FVC) > 0.7. COPD patients met GOLD criteria for the diagnosis of COPD, had a smoking history of ≥ 10 pack years and were recruited during the stable state defined as no exacerbations in the 4 weeks prior. Mild-moderate COPD patients were recruited with an FEV_1_/FVC ratio < 0.7 and an FEV_1_% predicted > 50%. COPD patients were not included if they were using maintenance antibiotics or oral corticosteroids, or had a diagnosis of other respiratory diseases other than COPD. All participants provided written informed consent (protocol reference: 05/Q1402/41).

### Nutritional Assessment

Protein and energy intakes were assessed using the EPIC-Norfolk Food Frequency Questionnaire (FFQ) [6] and a 7-day estimated food diary (FD), an average of the two methods were reported. Dietary intake data was analysed using the FFQ EPIC Tool for Analysis (FETA) (version 2.53) [7] and Nutritics © (version 6.0) [8], for FFQ and FD, respectively. FD and FFQ were monitored to ensure completeness and daily compliance with FD. Proportion of participants not meeting protein and energy requirements were calculated using PENG guidelines [9]. A threshold of 75% was used to define individuals not meeting the minimal recommended requirement for energy and protein, based on ESPEN guidelines [10].

### Anthropometric Measurements

Anthropometric measures were assessed, with body composition analysed using bioelectrical impedance analysis (BIA). Body mass index (BMI), waist to height ratio (WHtR) and FFMI were measured, with low FFMI defined as (a) < 15 kg/m^2^ for females and < 17 kg/m^2^ for males [2] or (b) adjusted for age, sex and BMI [11].

### Functional Capacity

Functional capacity was assessed using Short Physical Performance Battery (SPPB: standing balance, 4-m gait speed (4MGS), and five-repetition sit-to-stand motion (5STS)), 6 Minute Walk Test (6MWT), Timed Up and Go (TUG) and Handgrip Strength (HGS).

### Statistical Analysis

Comparisons between groups were assessed using independent t-tests, Mann–Whitney U tests, or Chi-squared tests, as appropriate.

## Results

### Subjects

21 COPD participants and 15 HNS were recruited. COPD patients had a median age of 58.0 years and mean FEV_1_ of 74.8% predicted (Table 1). 47.6% of COPD patients were current smokers with a median smoking history of 30.8 pack years. Approximately 50% of patients were using inhaled corticosteroids as part of triple therapy combinations. HNS self-reported were a median age of 57.0 years, with a mean FEV_1_ of 104.0% predicted. Groups were well matched for age and sex, and as expected COPD patients had a significantly lower FEV_1_% predicted and FEV_1_/FVC ratio.

**Table 1:** Clinical characteristics of COPD patients and healthy non-smoking individuals

Characteristics	COPD (n = 21)	HNS (n = 15)	*p*-value
Sex (M/F)	10/11	8/7	0.74
Age	58.0 [55.0–63.0]	57.0 [50.0–60.0]	0.13
Smoking status (current n, %)	10 (47.6)	N/A	N/A
Pack years	30.8 [22.0–34.5]	N/A	N/A
Exacerbations (prev 12 m)	0.95 (2.2)	N/A	N/A
0 (n, %)	9 (42.9)	N/A	N/A
1 (n, %)	6 (28.6)	N/A	N/A
≥ 2 (n, %)	6 (28.6)	N/A	N/A
FEV_1_ (L)	2.2 (0.5)	3.2 (0.8)	< 0.0001
FEV_1_% predicted	74.8 (14.0)	104.0 (12.2)	< 0.0001
FEV_1_/FVC ratio	57.1 (7.3)	77.1 (5.4)	< 0.0001
CAT (Total)	16.7 (8.1)	N/A	N/A
mMRC	1.0 [0.0–2.0]	N/A	N/A
SGRQ-C (Total)	33.0 (18.4)	N/A	N/A
Body composition			
BMI (Kg/m^2^)	27.2 [25.9–28.6]	27.9 [26.7–28.9]	0.68
Underweight (< 18.5)	1(4.8)	0 (0)	0.39
Normal weight (18.5–24.9)	3 (14.3)	2 (13.3)	0.87
Overweight (25–29.9)	14 (66.7)	11 (73.3)	0.67
Obese (≥ 30)	3 (14.3)	2 (13.3)	0.94
WC (cm)	94.8 [84.0–104.3]	92.0 [88.0–102.2]	0.76
Normal	4 (19.0)	3 (26.7)	0.94
High (> 94 (M) or 80 (F))	15 (71.4)	12 (73.3)	0.56
WHtR	0.57 (0.08)	0.55 (0.06)	0.54
Bioelectrical Impedance			
FM (Kg)	25.7 [17.5–28.5]	22.3 [16.8–30.4]	0.77
%FM	33.2 [26.6–40.4]	28.7 [20.4–41.8]	0.39
FFM (Kg)	47.1 [40.2–64.1]	50.4 [44.9–65.5]	0.28
%FFM	66.8 [59.7–73.4]	71.3[58.2–79.6]	0.39
FFMI (Kg/m^2^)	19.3 [15.9–20.5]	19.1 [17.1–21.2]	0.86
FFMI category (Franssen, 2014)			0.04
Normal	16 (76.2)	15 (100.0)	
Low	5 (23.8)	0 (0.0)	
FFMI category (Schols, 2014 (ERS))			0.22
Normal	19 (90.5)	15 (100.0)	
Low	2 (9.5)	0 (0.0)	
Nutritional intake*			
Nutritional requirements			
Energy requirement (kcal)	2424.0 [2084.0–2852.0]	2121.0 [1837.0–2553.0]	0.05
Protein requirement (g)	93.8 [82.9–111.1]	62.1 [58.6–68.2]	< 0.001
Food diary			
Energy intake (kcal)	1607.0 [1229.0–2237.0]	1926.0 [1642.0–2055.0]	0.32
Protein intake (g)	61.3 [49.8–88.9]	75.7 [60.9–100.3]	0.08
FFQ			
Energy intake (kcal)	1838.0 [1464.0–2557.0]	1830 [1622.0–2405.0]	0.81
Protein intake (g)	80.8 [62.1–95.0]	81.4 [69.7–101.8]	0.84
Average measure			
Energy intake (kcal)	1928.0 [1462.0–2121.0]	1899.0 [1803.0–2075.0]	0.64
Protein intake (g)	73.1 [63.8–85.8]	80.4 [73.0–95.0]	0.14
Energy requirements not met			
% of requirement met (energy)	79.1 [48.4–158.0]	85.6 [66.2–141.1]	0.43
% of requirement met (protein)	78.4 [53.6–163.9]	128.4 [88.5–200.3]	< 0.01
Individuals not meeting energy requirement	17 (85.0)	10 (71.4)	0.34
Individuals not meeting protein requirement	15 (75.0)	2 (14.3)	< 0.01
Proportion of individuals not meeting 75% energy requirement	10 (50.0)	4 (28.6)	0.21
Proportion of individuals not meeting 75% protein requirement	7 (35.0)	0 (0.0)	0.01

^*^1 COPD and 1 HNS did not return FD, therefore average measures intake was unavailable

### Body Composition

**Fig. 1:**
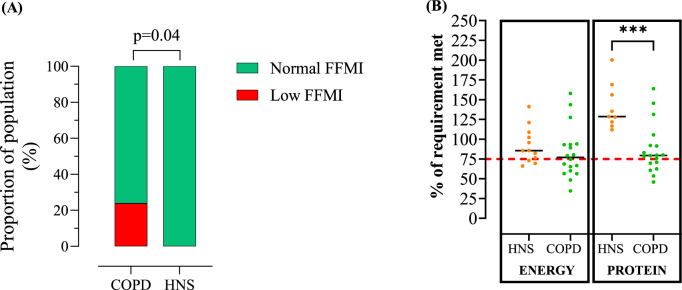
Comparison of COPD patients and HNS meeting normal FFMI criteria (**A**) and proportion of requirements met for energy and protein intake between COPD and HNS (**B**). Red line in panel B indicates 75% of nutritional requirement; maintaining intake ≥ 75% is associated with better outcomes in hospitalised COPD patients

An overweight BMI (> 25 kg/m^2^) was present in the majority of individuals in both groups with approximately 75% of participants demonstrating increased/high central adiposity defined by WHtR. There were no significant differences between COPD patients and HNS with regard to body composition (%FFM: 66.8 versus 71.3%; FFMI: 19.3 versus 19.1 kg/m^2^, respectively, Table 1). A numerically greater proportion of individuals with COPD had a low FFMI using ERS criteria compared to HNS (9.5 vs. 0.0%, *p* = 0.22), which was statistically significant when using age-sex-BMI adjusted criteria (23.8 vs. 0.0%, p = 0.04, Fig. 1a). There were 5 COPD patients with low FFMI using age-sex-BMI adjusted criteria; the BMI values ranged from 19.1 to 30.3 kg/m^2^, with 2 individuals being overweight and one classified as obese.

### Nutritional Intake

FD were not returned for 2 COPD patients and therefore did not contribute to average measures of protein and energy intake. Absolute median energy (1899.0 versus 1928.0 kcal, *p* = 0.64) and protein (80.4 versus 73.1 g, respectively, *p* = 0.14) intake were similar between COPD patients and HNS, respectively. A similar proportion of individuals in both groups were not meeting estimated total energy requirements (85.0% and 71.4% for COPD and HNS, respectively, *p* = 0.34). For estimated dietary protein requirements, significantly more COPD participants were not meeting 100% of requirements compared to HNS (75.0% versus 14.3% respectively, *p* < 0.01, Fig. 1b). Using the estimated requirement threshold of 75% [10], 35% of COPD patients did not meet their estimated protein requirements compared with 0% HNS (*p* = 0.01).

No differences in protein intake were observed between COPD patients with low *versus* normal FFMI.

### Functional Capacity

A moderate relationship was observed between total energy and protein consumption and functional capacity in COPD (SPPB: r = 0.57, *p* = 0.02 and r = 0.50, *p* = 0.049 for energy and protein, respectively), but not HNS. No relationship was observed for nutritional intake and other functional measures.

## Discussion

In this cohort of younger, mild-moderate COPD patients, we observed that a high proportion of individuals were overweight with excessive central fat deposition. The body composition of these COPD patients was not different to healthy individuals when using ERS criteria, as previously reported in mild-moderate COPD [12]. However, more COPD patients exhibited low FFMI, indicative of low muscle mass, when we used thresholds adjusted for age, biological sex and BMI. While total dietary energy consumption was similar between COPD patients and healthy individuals, protein intake was significantly lower in COPD patients, with 75% of individuals not meeting recommended daily requirements. These results identify a subgroup of younger COPD patients who display dietary insufficiency and reduction in skeletal muscle mass while being overweight.

The global prevalence of sarcopenia ranges from 10 to 27%, with variation due to classification systems and thresholds [13]. Sarcopenia is often missed when managing chronic conditions [14] as individuals may appear overweight. In our COPD cohort, sarcopenia was observed in 5 out of 21 individuals, with 3 being classified as either overweight or obese. The sarcopenic individuals identified here are likely at risk of further worsening of sarcopenia which is associated with reduced physical activity [14, 15].

Sarcopenic obesity naturally increases with age and is accelerated in chronic conditions such as auto-immune conditions and heart failure [16]. This may arise through various mechanisms including systemic inflammation directly causing muscle breakdown, physical inactivity causing disuse atrophy, and poor appetite [17]. These factors often interact with a reduction in FFM (metabolically active tissue) causing an increase in FM, further reducing the ability to maintain daily activity which in turn accelerates muscle mass loss in a vicious feedback loop [15]. Within the COPD cohort investigated here, approximately 24% of individuals exhibited evidence of sarcopenia. This suggests that COPD related factors such as inflammation, smoking and physical inactivity may contribute to more rapid sarcopenia development in a sub-group of relatively young COPD patients. Studies in older and more severe COPD patients reporting the presence of sarcopenia suggest that increasing years of malnutrition interact with disease related factors to cause accelerated sarcopenia [3].

It has been estimated that up to one third of COPD patients are obese [18, 19]. The obesity paradox is a concept derived from observations that overweight COPD patients have a reduced in-hospital mortality rate compared to underweight COPD, with the latter consistently associated with poor outcomes [19]. When malnutrition is integrated into the assessment of outcomes for hospitalised COPD patients, obese individuals who are malnourished suffer a significantly higher mortality rate and greater requirement for invasive mechanical ventilation, compared to COPD patients who are not malnourished [20].

Our findings highlight that sarcopenia exists in a subset of younger COPD patients, and we suggest that this is clinically important as sarcopenia can be easily missed in individuals who are overweight or obese. A cohesive multidisciplinary team approach to COPD care, which includes dietitians, can enable identification of such individuals coupled with appropriate nutritional intervention.

The most appropriate method for assessing sarcopenia in COPD is dual-energy X-ray absorptiometry (DEXA) scan alongside functional measures, therefore the use of BIA may be a limitation. However, BIA represents an accessible and less expensive alternative, which is widely used in clinical research settings. The study is cross-sectional in design and used a small sample size, limiting generalisability to wider COPD populations. A further limitation of this study is the lack of a control group of individuals who smoke but do not have COPD, this limits the assessment of disease *versus* smoking as contributory factors.

This study highlights that a subset of younger COPD patients are not meeting daily protein intake requirements and have sarcopenia. These findings suggest a potential opportunity for dietary intervention in younger individuals to prevent future sequalae of malnutrition.

## Data Availability

Data may be made available upon reasonable request.
